# ETHICS of AI Adoption and Deployment in Health Care: Progress, Challenges, and Next Steps

**DOI:** 10.2196/67626

**Published:** 2025-10-30

**Authors:** Obinna O Oleribe, Andrew W Taylor-Robinson, Christian C Chimezie, Simon D Taylor-Robinson

**Affiliations:** 1 College of Health, Human Services, and Nursing California State University Dominguez Hills Carson, CA United States; 2 Office of the Director General Nigerian Institute of Medical Research Lagos Nigeria; 3 Centre for Family Health Initiative Orange, CA United States; 4 College of Health Sciences VinUniversity Hanoi Vietnam; 5 VinUniversity-University of Illinois Smart Health Center VinUniversity Hanoi Vietnam; 6 Center for Global Health Perelman School of Medicine University of Pennsylvania Philadelphia, PA United States; 7 National Grid Bristol United Kingdom; 8 Department of Surgery and Cancer Faculty of Medicine Imperial College London London United Kingdom; 9 Department of Public Health Busitema University Mbale Uganda

**Keywords:** artificial intelligence, generative AI, ethics, equity, health care

## Abstract

Generative artificial intelligence (GenAI) is increasingly being integrated into health care, offering a wide array of benefits. Currently, GenAI applications are useful in disease risk prediction and preventive care, diagnostics via imaging, artificial intelligence (AI)–assisted devices and point-of-care tools, drug discovery and design, patient and disease monitoring, remote monitoring and wearables, integration of multimodal data and personalized medicine, on-site and remote patient and disease monitoring and device integration, robotic surgery, and health system efficiency and workflow optimization, among other aspects of disease prevention, control, diagnosis, and treatment. Recent breakthroughs have led to the development of reliable and safer GenAI systems capable of handling the complexity of health care data. The potential of GenAI to optimize resource use and enhance productivity underscores its critical role in patient care. However, the use of AI in health is not without critical gaps and challenges, including (but not limited to) AI-related environmental concerns, transparency and explainability, hallucinations, inclusiveness and inconsistencies, cost and clinical workflow integration, and safety and security of data (ETHICS). In addition, the governance and regulatory issues surrounding GenAI applications in health care highlight the importance of addressing these aspects for responsible and appropriate GenAI integration. Building on AI’s promising start necessitates striking a balance between technical advancements and ethical, equity, and environmental concerns. Here, we highlight several ways in which the transformative power of GenAI is revolutionizing public health practice and patient care, acknowledge gaps and challenges, and indicate future directions for AI adoption and deployment.

## Introduction

Artificial intelligence (AI), also referred to as augmented intelligence, currently plays multiple critical roles in public health and medical practice, the rapid implementation and profound impact of which were unforeseen just a few years ago [1-5]. The emergence of generative AI (GenAI) through the release of a popular large language model in late 2022 made AI readily accessible to the general population and brought transformational shifts in several sectors, including health care [6,7]. GenAI has changed how people interact with each other—how they communicate, exercise, work, do business, relate, and lead. As part of this societal seismic shift, GenAI is revolutionizing global health care systems.

Digital technology is fast becoming an integral part of public health and medical practices, providing validated tools for detecting, screening, diagnosing, caring for patients, and monitoring health-related parameters. GenAI has measurably improved patient care and enabled individuals to self-identify issues, thereby leading to better management of their health and well-being [8]. According to a 2025 survey of senior health care leaders, 95% of respondents believed that GenAI will transform the industry, with 85% of health care providers and 83% of “payer leaders” stating that it will “reshape clinical decision-making within three to five years” [9]. In total, 54% of all respondents reported that they were already seeing a meaningful return on investment in their organization after the first year of GenAI adoption.

The introduction of GenAI into public health and medical ecosystems offers enormous opportunities for training, research, patient care, and resource management [10]. Nevertheless, the potential benefits of AI are accompanied by profound ethical considerations and substantial implementation challenges. In this viewpoint, we contend that the effective adoption of AI within health care contexts is contingent upon systematically addressing these concerns. We further delineate recommendations intended to inform stakeholders seeking to foster the responsible development and deployment of innovative AI systems.

## Current Trends in Health Care

GenAI is currently used as a powerful tool to provide diverse services to health care and public health providers. This includes the delivery of personalized services to patients and accurate information to health care leaders, enabling them to improve the quality of services, efficiency, and effectiveness of care and to combat the increasingly widespread online dissemination of health misinformation and disinformation (Table 1).

**Table 1:** Common applications of artificial intelligence (AI) in public health and clinical medicine.

Application area	Role of AI	Current or expected benefits
Disease risk prediction and preventive care [11-13]	Predicts future susceptibility to many diseases using health records, lifestyle factors, and other data sources (eg, Delphi-2M and BlueDot).	Helps public health professionals plan. Has the potential to inform early interventions and long-term personalized risk estimates.
Diagnostics via imaging [14-16]	Supports automated detection of anomalies in medical imaging (x-ray, CT^a^, and MRI^b^) for tuberculosis, cancer, and other diagnoses.	Supports faster triage to reduce radiologist workload and ensures earlier detection with fewer missed cases.
AI-assisted devices and point-of-care tools [17,18]	Enables faster diagnosis by providing diagnostic insights in real time for cardiomyopathies and other abnormalities.	Supports faster diagnosis with potential use in nonspecialist settings, freeing specialists’ time and reducing delays.
Drug discovery and design [19-21]	Helps in identifying or designing new drug molecules, predicting toxicity, optimizing clinical trials, and repurposing existing drugs, thereby accelerating the drug development process.	Reduces time and cost of drug development compared to the traditional drug discovery process, potentially compressing decades into months and saving billions of dollars.
Remote monitoring [22,23]	Helps with continuous collection and analysis of physiological and behavioral data for chronic disease management and early detection of signs and symptoms (eg, wearables for PGHD^c^).	Supports early detection of complications, reduces hospitalizations, promotes better disease management, and provides more proactive care.
Integrating multimodal data and personalized medicine [15,23]	Combines genomics, imaging, and EHRs^d^ to tailor treatments to patients’ specific needs.	Allows more precise treatments with the potential to reduce adverse reactions and achieve better patient outcomes.
Patient and disease monitoring [24]	Provides tools and devices that help track disease progression to detect early disease symptoms.	Supports care in nonclinical settings to improve patients’ quality of life through early detection and diagnosis.
Robotic surgery [23,25]	Uses GenAI^e^-based algorithms to improve precision and control during surgical procedures.	Improves the effectiveness and efficiency of surgical procedures by enhancing precision, reducing surgeon fatigue, and improving safety.
Health system efficiency and workflow optimization [15,23,26]	Automates administrative tasks, prioritization, and resource allocation.	Reduces delays in administrative tasks by improving allocation of resources, leading to cost savings and fewer preventable complications.

^a^CT: computed tomography.

^b^MRI: magnetic resonance imaging.

^c^PGHD: personally generated health data.

^d^EHR: electronic health record.

^e^GenAI: generative artificial intelligence.

## Current Gaps in GenAI in Health Care and Mitigation Strategies

AI is expected to improve health care outcomes by facilitating early diagnosis, reducing the medical administrative burden, aiding drug development, personalizing medical and oncological management, and monitoring health care parameters on an individual basis, thereby allowing clinicians to spend more time with their patients [27]. Although the integration of AI into health care has the potential to transform the industry, it also raises ethical, regulatory, and safety concerns [28]. AI can rapidly analyze large and complex datasets; extract tailored recommendations; support decision-making; and improve the efficiency of many tasks that involve processing data, text, or images [29]. As the operability of GenAI in public health and medicine advances, significant gaps remain. AI systems risk perpetuating or amplifying existing health disparities when trained on current available data, which are largely nonrepresentative and noninclusive in nature [30,31]. AI tools and resources also lack explainability and transparency, which undermines clinician trust and introduces legal and ethical issues in safety-critical care [32,33]. Data silos, poor data quality, and limited interoperability remain major technical and organizational barriers to AI integration and use across the health care sector [34]. Regulatory, governance, and evaluation frameworks for safe clinical deployment are often incomplete or inconsistent across jurisdictions. Furthermore, models can degrade in new settings (dataset shift), and routine monitoring and maintenance of deployed models are frequently inadequate [35-38]. Other challenges include environmental concerns, hallucinations, inconsistent outputs, and various forms of cultural insensitivities across models in health. Table 2 summarizes some of the current gaps in AI adoption in health care.

**Table 2:** Common gaps in artificial intelligence (AI) adoption in health care and possible mitigation strategies.

Gap in AI technologies	Clinical and public health implications	Mitigation strategies
Bias and fairness [30,31]	Unequal performance across race, sex, and socioeconomic groups may lead to harm and widened disparities. For example, pulse oximeters and some AI tools perform worse for individuals with darker skin tones, and biased AI models can reduce clinician accuracy.	Use only diverse, inclusive, and representative datasets. Apply fairness-aware ML^a^ techniques (eg, reweighting and adversarial debiasing). Ensure routine equity audits during deployment.
Explainability and transparency [32,39]	“Black box” models are difficult to interpret, making legal and ethical accountability unclear. The lack of explainability and transparency has been linked to adoption failure.	Develop interpretable models or integrate XAI^b^ methods. Implement regulatory requirements that make explainability in safety-critical systems a requirement. Co-design model explanations with clinicians and patients.
Data access, quality, and interoperability [34]	Poor data quality, fragmented EHRs^c^, and governance issues reduce model utility and transferability. To date, many institutions are unwilling or unable to share data, while legacy systems and nonstandard formats impede seamless integration.	Implement common data standards across systems. Establish secure health data–sharing frameworks protected from cyberattacks and misuse. Improve data curation pipelines and maintain audit trails.
Generalizability and reproducibility [36,40]	Models validated on narrow cohorts fail in new hospitals or populations, producing unsafe predictions (overfitting). When models move across sites, performance drops and reproducibility issues arise even in published models.	Ensure multisite validation across demographics. Establish open-source benchmarks and reproducible pipelines. Implement stress testing for the dataset shift.
Regulation, governance, and evaluation standards [35,37]	Inadequate regulatory frameworks delay safe adoption or allow the use of poorly validated tools in practice, as there are no consistent premarket or postmarket standards.	Update and operationalize AI-specific regulatory pathways. Require premarket validation and postmarket monitoring. Establish independent ethics boards to ensure objective oversight.
Continuous monitoring and model predictive maintenance [36,38]	As some deployed models degrade over time (drift) and clinical workflows change, continuous monitoring is necessary to detect safety issues early.	Establish continuous monitoring pipelines for model drift. Implement active learning and regular model retraining. Operationalize clear sunset policies for unsafe models.
Evidence-based clinical evaluation [27,28]	A lack of prospective clinical trials or robust impact evaluations exists for most published models, as most studies were retrospective.	Fund and conduct prospective clinical trials and RCTs^d^. Apply implementation science frameworks. Report findings following approved AI and scientific guidelines.
Data privacy, security, and governance [35,41]	Consent models for secondary AI data use are unclear, and several large health datasets are at risk of breaches.	Use privacy-preserving ML techniques. Implement strong cybersecurity frameworks in hospitals. Ensure transparent consent and governance systems.
Clinical workflow integration and usability [42]	Poorly integrated tools disrupt patient care, increase workload, and provide outputs that are not actionable.	Adopt human-centered design approaches. Conduct pilot testing in real-world clinical workflows. Integrate workflow with EHR systems to reduce burden.
Workforce skills and trust [42,43]	Clinicians and the public currently lack adequate AI literacy, amplifying distrust. Inadequate training and limited AI competency impede responsible and appropriate use.	Operationalize AI literacy programs for clinicians. Ensure transparent communication with patients. Clarify liability and responsibility guidelines.
Equity in public health contexts [29,30]	Current AI models may underrepresent populations considered marginalized and neglect social determinants of health, leading to misallocation of resources.	Use diverse, inclusive, and representative datasets. Proactively include populations considered marginalized in model development and training. Ensure routine equity audits during deployment.
Conflicts of interest and transparency [27]	Vendor opacity, commercial incentives, and limited independent validation bias evidence and deployment decisions.	Ensure commercial interests do not override patient and public safety. Ensure developers sign a conflict of interest declaration form.
Computational cost and environmental impact, including carbon footprint [44-46]	Training and operating large AI models consume substantial energy and water resources, contributing to climate change and straining sustainability goals in the health sector.	Develop better energy-efficient architectures. Use carbon accounting and offsetting in model classification. Optimize hardware efficiency across models.
Hallucination with fabricated outputs [47-49]	AI systems may generate incorrect or entirely false but convincing outputs, posing patient safety risks if used for diagnosis, clinical advice, or health communication.	Use RAG^e^ and grounding in trusted medical databases. Ensure use of human-in-the-loop verification. Build uncertainty estimation into outputs.
Inconsistent outputs (stochasticity and reproducibility) [50-52]	LLMs^f^ may produce different answers to the same query depending on prompt wording or repetition, undermining reliability in clinical decision-making.	Understand the limitations of AI in the patient care spectrum. Ensure use of human-in-the-loop verification. Ensure health care service providers such as physicians and nurses take full responsibility for patient safety regardless of the use of AI tools.
Cultural insensitivity and lack of contextual grounding [53]	AI models trained primarily on Western or English datasets may overlook cultural beliefs, practices, idioms, and health priorities of diverse populations, leading to alienation, mistrust, or unsafe advice.	Include culturally diverse datasets and annotations. Partner with local communities to ensure context-aware AI design. Train multilingual and multicultural AI systems.

^a^ML: machine learning.

^b^XAI: explainable artificial intelligence.

^c^EHR: electronic health record.

^d^RCT: randomized controlled trial.

^e^RAG: retrieval-augmented generation.

^f^LLM: large language model.

To address these gaps, it is essential to be deliberate and proactive in the design, development, and deployment of new AI models by ensuring representative data, appropriate validation, transparency, and prospective clinical evaluation before deployment; implementing continuous monitoring for dataset shifts, retraining policies, and incident reporting after deployment; and encouraging stakeholders to invest in interoperable data infrastructure, clinician training, clear regulation, and system-level equity assessments. This underscores the ethical imperative to integrate ethics into all stages of AI design, development, training, and deployment.

Although AI has tremendous potential in public and clinical health care, there is an urgent need to mitigate these challenges to effectively harness these benefits for more effective and efficient health care delivery systems. Leaders and health care providers must address all or most of the issues identified in Table 2. This is achievable, as there are documented steps for the design, development, and deployment of AI models that are largely free (or relatively free) of these challenges.

## Maximizing AI Opportunities in Health Care

To maximize the opportunities that AI provides, health care leaders, public health specialists, and providers must work with biomedical engineers, computer scientists, and AI experts to develop interoperable data solutions, address biases, and ensure equity and fairness. In developing and deploying the next generation of health care AI tools, they must build transparency and ensure the development of explainable GenAI tools that enhance health care providers’ trust in AI, thus improving its use and subsequent better patient-related decision-making. These modifications will promote responsible, appropriate, and ethical practices in health care. For example, GenAI-driven tools such as Woebot (Woebot Health) [54], AI-powered mental health chatbots [55], and wearable electrocardiogram (ECG) apps such as the Apple Watch (Apple Inc) ECG feature [56] demonstrate how AI is already transforming health care by improving accessibility, decision-making, and transparency in patient care and data delivery. AI-powered chatbots and virtual health assistants, such as Babylon Health (eMed), provide patients with 24/7 access to health care advice, symptom checks, and appointment scheduling [57]. Imagine what could happen if these tools were trained with inclusive datasets that greatly minimize or eliminate bias, improve generalizability, and are open to providers. Such unbiased tools will accelerate adoption and use, saving providers’ time, enabling opportunity for better provider-patient interactions, and enhancing the accuracy of diagnosis and treatment, as well as the safety of hospital procedures.

Creating platforms that ensure high-quality, interoperable data can significantly enhance GenAI applications in health care. This will facilitate seamless data integration across different systems. Currently, GenAI-powered wearable health monitoring devices such as Apple Watch (Apple Inc), Fitbit (Google Inc), and Garmin (Google Inc) include pedometers, blood oxygen sensors, pulse oximeters, and electrodermal activity sensors to monitor skin temperature and stress. These personally generated health data, when analyzed, can be used to predict potential health risks and encourage preventive measures. Most of these devices are stand-alone products. However, system interoperability can bridge the gap between real-time monitoring and clinical decision-making. For instance, the ECG feature of a smartwatch that leverages GenAI to detect heart health anomalies can enable remote patient monitoring and provide explainable alerts to both lay users and health care professionals. Such alerts will help the wearer and the physician make timely, informed decisions [58].

Ensuring tools are trained using representative datasets is essential to ensure that GenAI model outputs appropriately reflect the entire population. Standardizing data collection protocols can enable consistency to be achieved across sources. Developing tools that detect and measure levels of bias in AI models and incorporating fairness constraints during the development process may help reduce biases [59].

Focusing on developing explainable GenAI models can help build trust among clinicians and patients because such models enable users to trust and understand how decisions are made, thereby fostering transparency and accountability. Integrating GenAI into clinical decision support systems that assist health care providers at the point of care can improve decision-making and patient outcomes. For example, the AI-driven Woebot mental health chatbot provides users with clear explanations for its therapeutic recommendations [54]. When suggesting cognitive behavioral therapy exercises, it explains their evidence-based benefits, such as reducing anxiety by addressing unhelpful thought patterns [37]. Similarly, the Apple Watch’s ECG feature builds user confidence and empowers individuals by providing instantaneous, actionable information, while stored data offer clinicians detailed insights into detected irregularities [59]. These applications demonstrate the value of transparent AI in improving user engagement and trust.

Making AI tools more user centered and integrated into health system workflows is essential to ensure a good user experience. Such tools will also be able to provide real-time monitoring and early warnings for health challenges, such as cardiac issues, thereby facilitating early professional evaluation and reducing morbidity and mortality [58,60]. In addition, updating and streamlining ethical guidelines and regulatory frameworks for AI in health care that prioritize data privacy, inclusivity, and transparency will facilitate appropriate, responsible, and equitable use of AI technology [56,57]. To achieve this, health leaders and biomedical engineers must collaborate with policymakers and other dominant stakeholders [61].

Moreover, early exposure of future health care professionals to GenAI at secondary and tertiary levels of education is critical to producing an AI-astute health workforce for primary, secondary, and tertiary care. Therefore, it is imperative to incorporate hands-on training and practical application sessions into both graduate and undergraduate curricula so that future health care professionals can work seamlessly with GenAI tools and datasets. Simulation exercises, case studies, and project-based learning are pedagogical approaches that can be tailored to enhance practical understanding. To improve engagement and effectiveness, learning pathways should be customizable to meet the needs of individual users, thereby promoting AI literacy among the emerging health care workforce. Some companies currently offer excellent case studies for health care students to learn about GenAI applications. Similarly, several colleges and institutions have created new courses on AI at the graduate level. However, more courses should be created at the undergraduate level, especially in historically minority-serving institutions. Each program should consider the diverse backgrounds, expertise levels, and specialties of participants [61,62].

Fostering interdisciplinary collaboration through joint programs and projects will enhance stakeholders’ awareness of the potential and limitations of GenAI technologies and thus identify their most appropriate use. Interdisciplinary knowledge sharing between health care professionals, data scientists, biomedical engineers, policymakers, and computer scientists can accelerate the discovery of more innovative, appropriate, user-friendly, inclusive, and applicable solutions [61,62].

## Bridging Equity Gaps in AI Adoption and Use

The future of GenAI in health care is poised to be transformative, fundamentally altering the landscape of public health and clinical practice. Similar to wielding a hammer, GenAI in the hands of trained and experienced health care providers will augment, rather than replace, skilled and experienced operators. It is a force for positive change when appropriately developed, modeled, and used properly. Making GenAI available to all individuals, irrespective of ethnicity, race, gender, or socioeconomic status, will reduce inequity and improve health outcomes. However, the current adoption and use of GenAI is not equitable across the health care industry, as large systems in high-income countries have unhindered access, while small organizations struggle to afford the tools they need most. Similarly, AI penetration in low- and middle-income countries remains limited due to inadequate infrastructure and insufficient financial resources. First-generation scholars and students from populations considered historically marginalized are also behind in AI adoption and use.

Health care systems adopting GenAI must prioritize people over profits to prevent inequity and its associated adverse outcomes, as the integration of advanced machine learning algorithms and big data analytics is not merely a trend but a paradigm shift that promises to enhance public health, improve clinical decision-making and patient experiences, and address systemic inefficiencies in resource allocation. To leverage these technological advances equitably and effectively, several key issues must be considered.

First, limited engagement by key stakeholders on how best to embed GenAI into health care provision poses a significant risk [63]. This challenge is exacerbated when medical, nursing, and allied professions are excluded from conversations that potentially impact health care services and professional practices. Establishing formal GenAI leadership roles will help ensure ethical and equitable use [51]. Health care leaders must drive the ethical and equitable integration of GenAI into health care services and ensure proper oversight to promote holistic, patient-centered, and professional care [64]. They can achieve this through a deliberate proactive leadership approach that thinks, plans, provides, processes, and communicates ahead to ensure seamless and timely transition of health care systems from a pre-GenAI era to one that is fully GenAI integrated [65,66].

Second, as the success of GenAI in health care depends on acceptance by both patients and providers, transparent communication about the benefits and limitations of GenAI, as well as demonstrations of its value, is essential for building trust. Our recent studies have revealed that very few employees are aware of the process, cost, and implications of GenAI adoption in their organizations [67,68]. This is worse for minority populations and underserved communities. Thus, proper and timely communication systems must be developed, adopted, and operationalized in accordance with the deliberate proactive leadership approach [66].

Third, to translate AI research into clinical practice across all populations, there is an urgent need for system-wide AI education, including a professional development component tailored to local contexts, with emphasis on underserved communities. Limited access to resources, including skilled and equipped AI trainers and the required infrastructure, hinders such on-the-job training. Therefore, there is a need to develop and popularize both accredited instructor-led and self-directed learning courses that provide introductory content on AI [69], as its opacity limits widespread adoption. Furthermore, as the complexities of GenAI and its implementation can negatively impact its use in health care practice [70], identifying discrepancies in priorities between health care managers and GenAI developers will lead to better collaboration. For instance, the development of GenAI applications with inclusive data that focus on health care leadership and management priorities should be a unified goal for all stakeholders [71]. These innovations must incorporate both the in-out (from providers to industry) and out-in (from industry to providers) approaches, placing providers and industry at the center of innovation, development, and deployment of new GenAI tools [61].

Finally, as much of the early adoption of GenAI has been concentrated in better-resourced provider settings, such as hospitals, academic medical centers, and large health system networks, deliberate steps must be taken to overcome barriers such as data infrastructure, technical capacity, investment, governance, and risk management, which tend to disproportionately impact resource-limited settings [72-74]. In the United States, for example, this gap is especially apparent in resource-limited settings, such as essential community providers, including federally qualified health centers, tribal or urban Indian clinics, and community or free clinics, which serve underserved populations in medically disadvantaged areas. Essential community providers face well-established challenges, including limited resources and health information technologies, and they exhibit lower rates of deployment of advanced digital tools compared to private systems [75]. The adoption of GenAI by community clinics and hospital departments should be supported financially and otherwise by governments and relevant foundations. Some countries, such as Vietnam, are ahead of the curve in this regard, illustrating how GenAI could enhance service efficiency, improve outcomes of interventions, and raise the quality of care provided by the health care industry [76,77]. Efforts to expand the availability of GenAI applications to underserved health care units across regions should be intensified, and the global health care community should also collaborate to ensure that further GenAI developments are tailored to address identified needs.

## Conclusions

GenAI technologies have the potential to transform health care by improving public health practices, enhancing diagnostic accuracy, personalizing treatments, automating services, and increasing administrative efficiency. Future developments in GenAI should be guided by the need to address health care’s most pressing AI-related challenges, especially environmental concerns, transparency and explainability, hallucinations, inclusiveness and inconsistencies, cost and clinical workflow integration, and safety and security of data (ETHICS). Similarly, AI regulation, governance, and clinical validation processes should be streamlined and strengthened to ensure the responsible and effective integration of AI in health care settings. Priority should also be given to establishing appropriate leadership and management structures and developing interoperability of data systems. By ensuring fairness, ethical practices, and appropriate educational and infrastructural initiatives, the global health community can strengthen the positive impact of GenAI, driving more efficient health care delivery systems and leading to improved patient outcomes.

## Abbreviations

AI: artificial intelligence
ECG: electrocardiogram
ETHICS: environmental concerns, transparency and explainability, hallucinations, inclusiveness and inconsistencies, cost and clinical workflow integration, and safety and security of data
GenAI: generative AI
